# The Amsterdam Sexual Abuse Case: What Scars did it Leave? Long-Term Course of Psychological Problems for Children Who have been Sexually Abused at a Very Young Age, and their Parents

**DOI:** 10.1007/s10578-020-01067-5

**Published:** 2020-10-03

**Authors:** Vionna M. W. Tsang, Eva Verlinden, Esther M. van Duin, Jos W. R. Twisk, Sonja N. Brilleslijper-Kater, Maj R. Gigengack, Arnoud P. Verhoeff, Ramón J. L. Lindauer

**Affiliations:** 1 Department of Child and Adolescent Psychiatry, Amsterdam UMC, University of Amsterdam, Amsterdam, The Netherlands; 2 Department of Social Pediatrics, Child Abuse and Neglect Team, Amsterdam UMC, University of Amsterdam, Amsterdam, The Netherlands; 3 Department of Epidemiology, Health Promotion & Healthcare Innovation, Public Health Service of Amsterdam (GGD Amsterdam), Amsterdam, The Netherlands; 4 Department of Clinical Epidemiology and Biostatistics, Amsterdam UMC, VU University, Amsterdam, The Netherlands; 5 De Bascule, Academic Center for Child and Adolescent Psychiatry, Amsterdam, The Netherlands; 6 Department of Sociology, University of Amsterdam, Amsterdam, The Netherlands

**Keywords:** Child maltreatment, Sexual abuse, Infants, Preschoolers, Post-traumatic stress disorder

## Abstract

Longitudinal research of CSA in infancy and early childhood is scarce. The current study examined the long-term course of psychological outcomes (PTSD, dissociation and internalizing and externalizing behavioral problems) in children who were sexually abused in the early childhood. Additionally, we looked into the outcomes for their parents by assessing PTSD symptoms and negative emotional reactions towards the sexual abuse of their child. We examined the outcomes for five consecutive years in a sample of children (n = 45) who were sexually abused at a very young age (0–3) and their parents (n = 42), included in the Amsterdam Sexual Abuse Case-study. We found that outcomes following CSA in early childhood go beyond PTSD symptoms and can manifest in atypical symptoms such as behavioral problems. Parents experienced persistent PTSD in the years following CSA disclosure. CSA in very young children warrants long-term monitoring, as negative outcomes still present 8 years later.

**Electronic supplementary material:**

The online version of this article (10.1007/s10578-020-01067-5) contains supplementary material, which is available to authorized users.

## Introduction

Child Sexual Abuse (CSA) occurs in all countries, all cultures, all socioeconomic statuses and at all ages, even the youngest imaginable, such as infants. CSA is one of the most severe forms of trauma, owing to its possible adverse outcomes [1], and is an even stronger and more consistent predictor for mental health problems than physical abuse [2].

CSA includes a range of sexually transgressive activities. This diversity alone leads to myriad problems in the further development [3]. Studies indicate that CSA might negatively affect children’s psychological well-being and development [4], that could result in PTSD, dissociation or behavior problems [3, 5, 6]. With regard to behavior problems, generally, differentiation has been made between internalizing and externalizing problem behavior in developmental psychopathology studies [7]. Externalizing problem behavior is defined as actions outwardly, such as aggression, conduct problems, oppositionality and hyperactivity, whereas internalizing problem behavior is defined as processes within oneself, such as depression, anxiety, and somatic problems [8].

Research on these negative outcomes of CSA are primarily focused on children abused at an age of seven years and older. This while an estimate of 25–35% of CSA cases concern children below the age of seven [3, 9]. There is a mistaken belief that young children who experience trauma will be unaffected because they are cognitively too immature to understand or recall the traumatic experience [10, 11]. Generally, the earlier in life a traumatic experience occurs, the less specific and more pervasive the negative outcomes seem to be [12]. In these early years of life, the brain is being physically formed by environmental influences. Thus, the impact of severe stress and trauma can leave an ineffaceable imprint on the brain [12, 13]. Therefore, infants and young children seem to be even more vulnerable to traumatic stress than older children and adults [12].

CSA does not only affect the child, but could also emotionally affect their parent(s). In the aftermath of CSA disclosure, parents too, may develop psychological disorders, such as depression and PTSD [14–17]. Additionally, a study of Dyb et al. [14], in which a CSA case at a daycare center has been examined, shows that CSA related events (e.g. testifying in court, hearing the verdict, and media exposure) also contribute to a significantly elevated level of stress for parents. It is relevant to look into the outcomes for parents, given their crucial role in the recovery of their child following sexual abuse [18], especially when the child is at an early developmental stage in which they are fully or mostly dependent on their caregiver.

Although literature in CSA has widely broadened the last few decades, a closer look into the literature in CSA outcomes, reveals a number of methodological shortcomings, namely a lack of juridical proof of the CSA, the co-existence of other forms of abuse, the possible influence of treatment (on natural recovery) [19], and the diverse samples (e.g. clinical or national prevalence samples) [20]. Additionally, most studies are retrospective, which may lead to recall biases [21]. Moreover, nondisclosure is a persistent problem in CSA and many CSA cases remain unrecognized [4], especially in young children with limited verbal capacities [22].

Despite the growing literature in negative sequelae following CSA, studies that have specifically looked into children who have been sexually abused in infancy or (very) early childhood, are still rare. Our previous study [17] examined the psychological sequelae three years after disclosure and indicated that outcomes were more salient in behavior problems, and less in PTSD. This conclusion was based on one time point, and leads to the question how these outcomes develop on longer term. However, knowledge on the long-term course of CSA in very young children is still insufficient. In order to be able to offer and improve support for children and their parents, it is important to gain knowledge on the long-term outcomes following sexual abuse in infancy or early childhood.

The Amsterdam Sexual Abuse Case [ASAC; 23] enables us to study the long-term consequences for victims sexually abused at a very young age without most of the previously mentioned methodological limitations. To date, the ASAC is the largest juridical proven CSA case in worldwide history with one convicted perpetrator. In late 2010, this CSA case with an exceptional magnitude came to light during a child pornography investigation in the USA. It led to the discovery that in Amsterdam, the Netherlands, a male daycare employee, who was also working as a babysitter, possibly sexually abused 150 infants and toddlers. The ASAC-study is unique owing to the homogeneity of the group, namely; one convicted perpetrator, extrafamilial CSA, the exceptionally young age of the victims, juridical proof, detailed documentation of the sexual abuse, and the absence of other forms of child maltreatment [17].

The current study is a part of the ongoing ASAC-study [24] and aims to investigate the longitudinal consequences of sexual abuse that took place in infancy and early childhood. We addressed the following research questions regarding children: (1) What is the psychological long-term course for children who have been sexually abused at a very young age, in terms of PTSD symptoms, dissociation, and behavioral problems, and how do symptoms change over time? The following research questions regard parents: (2) What are the long-term consequences for parents following the disclosure of the sexual abuse in their child in terms of PTSD symptoms and negative emotional reactions towards the sexual abuse of their child and how do symptoms change over time?

## Methods

### Participants

As mentioned briefly, a man was suspected of sexually abusing over 150 infants and toddlers. The suspect confessed to the abuse of 87 young children and was prosecuted and sentenced for the sexual abuse of 67 children as parents of 20 children decided to keep their child out of the court records, due to privacy matters. Immediately after disclosure, an emergency outpatient clinic was set up to examine the children physically and psychologically [25, 26].

The current study examined the course of these sequelae for victims and their parents annually over five consecutive years, starting three years after the disclosure of the ASAC (2013–2017). The ASAC study protocol is available for perusal on https://bmcpsychiatry.biomedcentral.com/articles/10.1186/s12888-014-0295-7.

Parents and children who have been involved in the physical and psychological examination right after the disclosure, or parents who have been in contact with the Public Health Service of Amsterdam as part of the aftercare, were contacted by the second author (EV) and asked if they wished to receive information about participation in a longitudinal study. Certainly, additional questions would be answered in mean time. After parents decided to participate, written consent was requested. Informed consent was asked separately for the examination of the police reports and to obtain information about the psychological help parents and children received.

Inclusion criteria were as follows: that parents and the child(ren) took part in the physical and psychological examination or that parents had been in contact with the Public Health Service of Amsterdam as part of the aftercare program. Secondly, an inclusion criterion was that the child sexual abuse had been confirmed or highly suspected.

The sample included parents of confirmed victims and suspected victims. A child was considered a confirmed victim, in case the perpetrator had confessed to the sexual abuse and/or pornographic imagery of the child was found. A child was considered a suspected victim, in case the child had been in direct contact with the perpetrator, in daycare or babysit setting, and when the parents highly suspected sexual abuse in their child. These children were included in the current study on the grounds that the alleged CSA could not be ruled out, and since the current study also served another purpose, namely the monitoring of the well-being of victims and their parents. Therefore, we considered it unethical not to allow these children (n = 8) and their parents to participate. Also, the first follow-up study has shown that there are no significant differences in outcomes between the groups confirmed victims and suspected victims [17].

In total, 42 parents participated in at least one of the five time points reporting on their own psychological functioning and the psychological functioning of their child(ren) (n = 45). The full overview of the participation rate is presented in the flow chart of our previous study [17]. The age of the children at abuse onset was between 0 and 3 years. The demographic characteristics and characteristics of the CSA are presented in Table 1.

**Table 1:** Demographics and characteristics of the abuse

	Mean (min-max)		SD	
Age children at abuse onset (y)	1.4 (0–3)		0.9	
Age children at first assessment (y)	6.2 (3–9)		1.3	
	*N*		*%*	
Gender child (male)	30		66.7	
*Ethnicity child*				
Native Dutch	45		100	
*Ethnicity participating parents and their partners*	*Mother*	*Father*	*Mother*	*Father*
Native dutch	30	33	66.7	73.3
Non-native western	9	6	20.0	13.3
Non-native non-western	5	5	11.1	11.1
Unknown	1	1	2.2	2.2
*CSA type (n confirmed victims* = *37)*				
Exposure of genitals to child	31		83.8	
Ejaculation onto child	25		67.6	
Fondling^a^	34		91.9	
Oral copulation	21		56.8	
Penetration of anus or vagina with finger, penis or sex toy	13		35.1	
*Frequency*				
Once or twice	16		43.2	
Three to ten times	15		40.5	
More than ten times	4		10.8	
Unknown	2		5.4	
Cases with pornographic evidence	15		40.5	
*Location of abuse*				
Daycare	13		35.1	
Home	20		54.1	
Both	4		10.8	

Abuse characteristics were obtained from police reports. First assessment is 3 years after disclosure of the abuse.

^a^Described by perpetrator as touching genitalia or masturbating the child.

### Procedure

The data for this longitudinal study were gathered during five annual time points between 2013 and 2018. Parents were contacted by the researchers to plan a face-to-face appointment at the Amsterdam University Medical Center, the Public Health Center Amsterdam, or at parents’ home if preferred by parents. Secondly, they were asked to fill out online questionnaires about their child and themselves. The questionnaires were sent from a secured online environment and could be filled out at home. After each time point, parents received an individual report of the outcomes, including a personalized advice in potential worrisome outcomes from professional perspective. We also offered consultation according to the needs of parents. Alteration in the participation, retraction, and inflow was possible during each time point. Parents did not receive financial incentives for participation, but could apply for travel allowance.

Due to the sensitivity of the topic and risks of media exposure, we have tried to keep the researchers involved in this study as consistent as possible. The researchers involved in this study are all educated as child psychologists and experienced as researcher or clinical worker in the trauma field. For the same reason, no interns, research assistants or external parties have been in contact with the parents. During the five time points, the attrition rate was very low. Parents of five children dropped out after T1 and parents of two children dropped out after T4. Dropout is defined as parents who withdrew their informed consent for further participation in the study.

### Measures

Since the current study is a part of the ongoing longitudinal ASAC-study [24], we aimed to ensure the continuation of previous assessments by using the same instruments. This results in a big overlay in instruments. This paper reports the results of the questionnaires administered to parents only. The measures described below were employed in the current study. For a detailed description of these measures, supplementary material is available online or on request.

To assess psychopathology in children, we have used the following instruments. The Dutch version [27] of the Children’s Revised Impact of Event Scale, Parent Version [CRIES-13; 28] was used to measure PTSD symptoms in children. The 13 items asses symptoms of intrusion, avoidance, and hyperarousal. The total score indicates the level of severity of the posttraumatic stress response, where a total score of 30 or higher was used as clinical cutoff [29]. The Child Dissociative Checklist [CDC; 30], Dutch version [31] was used to measure dissociation symptoms in children. The total score could range from 0 to 40. Scores of 12 and higher were considered clinical, indicating pathological dissociation. The Child Behavior Checklist 1½-18 [CBCL; 32], Dutch version [33] was used to determine the level of internalizing and externalizing behavioral problems in children. The questionnaire consisted of 100 (for children from 1½–5 years old) to 113 items (for children from 6–18 years old) on 3-point Likert scale from 0 (not true) to 2 (very true or often true). T-scores were calculated for each subscale. A T-score of 65 or higher indicates problem behavior at clinical level.

To assess psychopathology in parents, the following self-report instruments were used.

The Impact of Event Scale-Revised [IES-R; [34], Dutch version [35] was used to determine PTSD symptoms in parents. The total score could range from 0 to 88, where a total score of 23 or higher was used as clinical cutoff. Lastly, the Parent Emotional Reaction Questionnaire [PERQ; [36], Dutch version [37] was used to measure the negative emotional reactions of parents towards the sexual abuse of their child. The total score ranges from 15–75 and results in three subscales: distress, shame, and guilt.

### Psychological Support

Besides, we collected the psychological treatment data, given that many parents and children received psychological support after the disclosure of the ASAC and because of the indicated influence of psychological treatment on the outcomes. In this paper, we use the term psychological support for both psychological help and treatment.

As psychological support varied from one or two appointments of parental advice to years of intensive psychotherapy, and was carried out by diverse mental health care centers, it was important to categorize it in order to gain more insight. Categorizing was done in the following steps. Initially, the first author (VMWT) summarized the files, for children and parents, each by time point. Secondly, four mental health professionals: NS (clinical psychologist), IH (child psychiatrist), KZ (healthcare psychologist) and EB (healthcare psychologist) scored the cases using the secured online system. For each case, the question was: ‘In which category does this case belong?’ The answer options were: a light form, a moderate form, or an intense form of psychological support. Scores were based on the combination of clinical judgement, frequency of treatment and level of care (e.g. preventive care, primary care, specialized care). Consensus was defined as an unanimous score, or when three out of four professionals agreed. The cases that did not reach consensus (psychological support child: 21.3%, psychological support parents: 14.9%) were categorized by the principal investigator, an expert in childhood trauma (RJLL), based on his own clinical experience and the reasoning of the mental health professionals.

### Ethical Considerations

The ASAC-study was approved by the Medical Ethics Review Committee of the Amsterdam University Medical Centers, location Academic Medical Center. Informed consent was obtained from all participating parents included in the study.

### Data Analysis

The questionnaire data and the descriptive statistics were analyzed using IBM SPSS Statistics, version 25. Linear mixed model analyses (for continuous outcome variables) and logistic Generalized Estimating Equation (GEE) analyses (for dichotomous outcomes) were used for the longitudinal analyses. Mixed model analyses and GEE analyses were used in order to take into account the dependency of the repeated observations within the child or parent [38]. A p-value of < 0.05 was considered statistically significant. Both mixed model analyses and GEE analyses were performed with STATA, version 15.

## Results

### Descriptive Statistics

Over the five time points, 71.1% of the children (n = 32) and 66.7% of the parents (n = 30) received some form of psychological support. In 33.3% (n = 10) of the cases, the psychological support considered both parents, in some 50.0% (n = 15) of the cases it considered individual support for the mother, and in 16.7% (n = 5) of the cases the father.

Table 2 shows the percentages of clinical symptoms for children and parents at each time point. Over the five time points, an average of 4.6% of the children showed clinical PTSD symptoms and 2.0% of the children showed clinical dissociation. As shown in the Table 2, the percentages of clinical scores on PTSD symptoms seemed to fluctuate over time, while the percentages on dissociation seem to remain stable and eventually decrease.

**Table 2:** Psychopathology in children and parents

Years after CSA	T1	T2	T3	T4	T5	M
	(clinical %)	(clinical %)	(clinical %)	(clinical %)	(clinical %)	(clinical %)
*Questionnaires children*						
PTSD (CRIES)	4.6	5.0	2.6	2.6	8.3	4.6
Dissociation (CDC)	2.3	2.5	2.6	2.6	0.0	2.0
Total behavior problems (CBCL)	11.4^b^ 15.9	10.0^b^ 22.5	10.3^b^ 10.3	12.8 ^b^ 10.3	13.9^b^ 11.1	11.7^b^ 14.0
Internalizing behavior problems (CBCL)	6.8^b^ 27.3	10.0^b^ 27.5	18.0^b^ 12.8	5.1^b^ 23.1	2.8^b^ 30.6	8.5^b^ 24.3
Externalizing behavior problems (CBCL)	15.9^b^ 13.6	5.0^b^ 20.0	0.0^b^ 18.0	5.1^b^ 12.8	5.6^b^ 8.3	6.3^b^ 14.5
*Questionnaires parents*						
PTSD (SVL)	25.0	20.0	18.0	10.3	19.4	18.5

CBCL scores are gender− and age−standardized.

*M* mean clinical percentages over five time points.

^b^*Subclinical.*

**Fig. 1:**
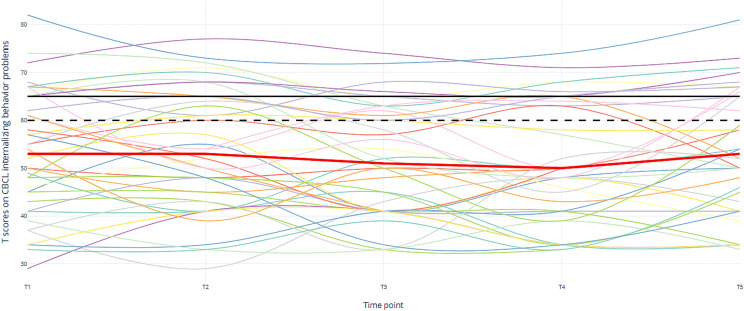
Individual trajectories of children on internalizing problem behavior (CBCL). The black line indicates the clinical cut-off. The dotted black line indicates the sub-clinical cut-off. The red line indicates the group based mean T-scores over time

Overall, clinical scores (Table 2) on internalizing problem behavior were higher (*M* = 24.3%) than clinical externalizing problem behavior (*M* = 14.5%). Figure 1 illustrates the individual trajectories of internalizing problem behavior over five time points. Each colored line represents the individual trajectories of the children. The striking observation is that while the percentages at each time point may not differ much, the individuals making up this percentage differ per time point.

As we can see in the table in Table 2, 26.7% (*M* = 18.5%) of the parents scored at a clinical level of PTSD symptoms at least at one of the five time points. The PTSD symptoms in parents fluctuated over time. There are no clinical cut-off points nor subscales available for the PERQ (negative emotional reactions towards the CSA of their child) yet and therefore these are excluded from Table 2.

### Longitudinal Analyses: The Development Over Time for Children

For the descriptive statistics, we used data of the five time points in order to include the data of all children and their parents. For the longitudinal analyses, we used the years after CSA as a time variable, because the ages of the children vary widely. Secondly, years after CSA is more informative and relevant in the study of the consequences following CSA than the year of measurement. Thirdly, this way it allows us to map out the consequences of CSA on a longer term than time points alone. We used the group ‘5 years after CSA’ as reference category because this group is significantly larger than the group ‘4 years after CSA’ and therefore provides more power. Since the current study regards an observational longitudinal study, we believe the importance of not only reporting statistical significances, but to focus on the development over time as well. In all analyses in outcomes for the children, we took the severity of the abuse and psychological support the child received into account as covariates.

The mixed models analysis (Table 3) was used to assess PTSD as outcome variable for children. For PTSD in children, the scores seem to increase until 7 years after CSA, and thereafter decrease at 8 years after CSA. No significant differences were observed between the years, indicating no clear increase or decrease in PTSD symptoms over the years. Table 4 shows the results with dissociation as outcome variable. There is a slight decrease over the years, yet not significant.

**Table 3:** Results from mixed models of changes in PTSD in children

N years after CSA (ref = 5 years)	b (SE)	CI	
4	−.14 (1.89)	−3.55	3.84
6	.24 (1.71)	−3.11	3.58
7	1.50 (1.70)	−1.82	4.82
8	.15 (1.86)	−3.49	3.79

Adjusted for sexual abuse severity and psychological treatment child.

**Table 4:** Results from mixed models of changes in dissociation in children

N years after CSA (ref = 5 years)	b (SE)	CI	
4	.13 (.50)	−.85	1.12
6	−.51 (.44)	−1.37	.36
7	−.34 (.44)	−1.20	.52
8	−.85 (.48)	−1.80	.09

Adjusted for sexual abuse severity and psychological treatment child.

As regards behavioral problems, the results of the mixed models analyses for internalizing, externalizing, and total behavior problems are shown in Table 5. In internalizing problem behavior, the scores were overall decreasing over the years, with a significant decrease 8 years after CSA (*b* = −4.30, SE = 1.53). The externalizing behavior problems were also decreasing generally, and significantly from 7 years after the CSA (*b* =−3.33, SE = 1.64) and 8 years after the CSA (*b* = −6.10, SE = 1.80). Regarding total behavior problems, we found a significant decrease over the years from 6 (*b* = −2.81, SE = 1.32) to 8 years (*b* = −6.15, SE = 1.45) after the CSA.

**Table 5:** Results from mixed models of changes in clinical behavioral problems in children

	*b (SE)*	*CI*	
*Internalizing behavioral problems*			
N years after CSA (ref = 5 years)			
4	−2.05 (1.55)	−5.09	.98
6	−1.69 (1.40)	−4.43	1.05
7	−2.54 (1.39)	−5.27	.19
8	−4.30 (1.53)**	−7.30	−1.31
*Externalizing behavioral problems*			
N years after CSA (ref = 5 years)			
4	−1.62 (1.82)	−5.20	1.95
6	−2.33 (1.65)	−5.56	.91
7	−3.33 (1.64)*	−6.54	−.13
8	−6.10 (1.80)**	−9.62	−2.58
*Total behavioral problems*			
N years after CSA (ref = 5 years)			
4	−1.92 (1.46)	−4.79	.95
6	−2.81 (1.32)*	−5.40	−.22
7	−4.04 (1.31)**	−6.62	−1.47
8	−6.15 (1.44)**	−8.98	−3.31

Adjusted for sexual abuse severity and psychological treatment child.

*p<.05, **p<.01.

Additional analyses were carried out using the Generalized Estimating Equations (GEE) to examine behavioral problems over time. The outcome behavioral problems was categorized into clinical or non-clinical. The GEE outcomes in internalizing and externalizing problem behavior were comparable to the outcomes of the mixed models analyses (see Table 6), both internalizing and externalizing behavioral problems were overall decreasing. Whereas regarding total behavioral problems, we found that it was slightly fluctuating, with a significant lower score 7 years (*OR*  0.30, 95% CI [0.12, 0.77]) after CSA, but also a significant increase 8 years (*OR*   0.41, 95% CI [0.18, 0.92]) after CSA.

**Table 6:** Results from GEE model predicting clinical behavioral problems in children

	*OR (robust SE)*	*CI*	
*Internalizing behavioral problems*			
N years after CSA (ref = 5 years)			
4	.69 (.27)	.32	1.50
6	.57 (.20)	.29	1.12
7	.46 (.17)*	.22	.94
8	.37 (.14)**	.18	.76
*Externalizing behavioral problems*			
N years after CSA (ref = 5 years)			
4	1.43 (.53)	.68	2.97
6	.83 (.31)	.40	1.73
7	.66 (.37)	.22	1.99
8	.36 (.21)	.12	1.13
*Total behavioral problems*			
N years after CSA (ref = 5 years)			
4	.56 (.29)	.20	1.57
6	.64 (.28)	.27	1.53
7	.30 (.14)*	.12	.77
8	.41 (.17)*	.18	.92

Adjusted for sexual abuse severity and psychological treatment child.

*p<.05, **p<.01.

### Longitudinal Analyses: The Development Over Time for Parents

Regarding parents, it was more relevant to study the outcomes in time points instead of years after the abuse, as the moment of disclosure was crucial for them. All analyses were adjusted for abuse severity and psychological support in parents.

PTSD in parents (Table 7) seemed to remain stable over the years. In addition, the mixed models analyses do not show a significant increase nor decrease in PTSD symptoms in parents. Table 8 shows the mixed models analyses we used to assess the negative emotional reactions of parents to the CSA of their child as outcome variable. In this outcome variable, there was a clear decrease in the negative emotional reactions of parents on the CSA. Significant lower scores were found at the time points 3 (*b* = −2.78, SE = 1.26), 4 (*b* = −6.28, SE = 1.26), and 5 (*b* = −5.11, SE = 1.27).

**Table 7:** Results from mixed models of changes in PTSD in parents

Ref = Time point 1	*b (SE)*	*CI*	
T2	−.05 (.05)	−.14	.04
T3	−.03 (.05)	−.13	.07
T4	−.13 (.05)*	−.23	-.03
T5	−.05 (.05)	−.15	.05

Adjusted for sexual abuse severity and psychological treatment parent.

*T* time point.

*p<.05.

**Table 8:** Results from mixed models of changes in parent’s emotional reactions to child sexual abuse

Ref = Time point 1	*b (SE)*	*CI*	
T2	−2.14 (1.18)	−4.46	.19
T3	−2.78 (1.26)*	−5.24	−.31
T4	−6.28 (1.26)**	−8.74	−3.82
T5	−5.11 (1.27)**	−7.60	−2.63

Adjusted for sexual abuse severity and psychological treatment parent.

*T* time point.

*p<.05, **p<.01.

## Discussion

In this study, we aimed to examine the long-term course of PTSD, dissociation and behavioral problems in children who were abused at a very young age, as well as PTSD and negative emotional reactions towards the CSA in their parents. To discuss the results, we return to the first research question posed at the beginning of this paper, namely: What is the psychological long-term course for children who have been sexually abused at a very young age and how do symptoms change over time?

On one hand, we observed low percentages of PTSD and dissociation in children at each time point. On the other hand, we found that, regardless of the severity of the sexual abuse and psychological support of the child, PTSD symptoms seemed to remain stable over time, even until 8 years after CSA. Furthermore, dissociation symptoms seemed to decrease over time.

The low number of children with clinical PTSD symptoms and dissociation we found is not surprising. As De Young and Scheeringa [39] suggest, this might be explained by the fact that the PTSD symptoms re-experience and avoidance were difficult to determine as the children were cognitively too young at the time of the traumatic event and therefore did not develop an autobiographic memory yet. In addition, avoidance symptoms and negative cognitions related to PTSD are deeply internalized reactions, making them hard to identify by another respondent than the child itself [39]. The slight fluctuation in PTSD symptoms over time we observed could be influenced by the disclosure of the sexual abuse to children. Some children have been informed about the sexual abuse over the years. The influence of disclosure will be examined in our ongoing study.

Regarding behavioral problems, nearly a quarter of the children exhibited internalizing behavioral problems and another 15% exhibited externalizing behavioral problems at clinical level. Over time, these behavioral problems decreased significantly. Despite this decrease, the number of children who exhibited behavioral problems at a clinical level kept fluctuating over time. This fluctuation might be explained by the interaction with PTSD symptoms. Milot et al. [40] found that PTSD symptoms might influence the development of internalizing and externalizing behavioral problems. However, to date, no longitudinal studies have focused on CSA in early childhood and the course of behavioral problems. It should also be noted that the studies we compared our study to, are not completely comparable as they differ in subjects, research setting and research design. Again, this emphasizes the gap in CSA literature studying children who have been sexually abused in infancy or very early childhood in a longitudinal study.

The high number of children exhibiting clinical behavioral problems is similar to our first follow-up study in which Van Duin et al. [17] found that 24% of the children exhibited internalizing problem behavior at a clinical level. Also, the findings of the current study tie well with what Beaudoin et al. [41] found in children who were sexually abused at an older age (3 to 6 years old), namely that 43% of them exhibited clinical internalizing problem behavior and 33% clinical externalizing problem behavior. These high percentages might indicate that preverbal trauma manifests into behavioral problems, than in trauma symptoms. However, it should be mentioned that both cited studies do not concern a longitudinal study design.

Regarding parents, sexual abuse in very young children led to clinical PTSD symptoms in 27% (*M* = 18.5%) of the parents at one or more time points. Over the years, PTSD symptoms in parents seemed to fluctuate, regardless of the CSA severity and psychological support for parents. This is in contrast to the clear drop in negative emotional reactions (e.g., fear, sadness, anger, shame, and guilt) in response to the sexual abuse of their child. The high number of parents with clinical PTSD symptoms accord with earlier findings in which nearly 20–30% of the parents suffered from PTSD symptoms three to four years after disclosure [14, 17].

It is interesting that the negative emotional reactions of parents towards the CSA decreased significantly, while PTSD in parents kept fluctuating. A likely explanation is that reactions as fear, sadness, and anger are primary emotions that are more observable and will surface more easily in response to a stressful or distressing event. PTSD, however, is a serious mental disorder in which the neurobiological system is affected and its symptoms are more pervasive. In addition, during the years following disclosure, parents were repeatedly confronted with triggers such as juridical procedures and media exposure. Dyb et al. [14] found that such triggers could increase the level of stress that parents experience. This might explain why PTSD symptoms did not decrease but seemed to keep fluctuating over time.

The current study has several strengths that need to be addressed. This longitudinal study design provides a unique perspective on the course of the symptomatology. Most previous studies have focused on outcomes at a specific age or point in time, a so-called snapshot approach [42]. The current study provides insight until eight years after the CSA and is ongoing. The value of our study also lies in the fact that we can study extrafamilial CSA without the confounding effects of other forms of child maltreatment, and the homogeneity of the group. Namely; one convicted perpetrator, juridical proof, detailed documentation of the sexual abuse, and the exceptionally young age at onset of CSA (0–3 years old). Lastly, we tested our significant outcomes of the mixed models analyses for behavioral problems in children a second time, using GEE analyses, which leads to stronger statistical evidence.

A number of potential limitations need to be considered as well. A major source of limitation is the relatively small group of participants. Therefore, we have carefully chosen specific statistical methods in order to draw reliable conclusions with such small groups. A second limitation is the lack of a control group. Nevertheless, the questionnaires we used were contrasted against norm groups and were corrected for age and gender, if available. Thirdly, the psychological assessments of the children were reported by parents. Presumably, the more covert problems like internalizing problem behavior, PTSD and dissociation may be harder to detect by parents and may differ if reported by the victims themselves. Research has shown that cross-informant agreement is typically low which makes it important to study different sources of information [43]*.* Moreover, only one of both parents filled out the self-report questionnaires. It could be the case that the most affected parent did not participate to avoid being confronted with the abuse of their child at each assessment. Another limitation related to the measurements is that we used the cut-off for the ages 8 to 18 in the CRIES because there is no cut-off for children of 8 years and younger available yet. Given that our findings are based on a limited number of participants, from a single sexual abuse case, implicates that these outcomes should be treated with considerable care.

Taken together, we have found that children who are sexually abused as infants and toddlers can experience negative psychological symptoms over the course of several years after CSA, even if, at the time of the abuse their cognitive capacity was limited. It is becoming clear that symptoms within children vary in atypical problems as behavioral problems, rather than only in trauma symptoms. This suggests we should not only focus on clear-cut trauma symptoms, but we have to be attentive of atypical trauma symptoms that might manifest in internalizing and externalizing behavioral problems. Furthermore, symptoms in children do not necessarily decrease over time despite the fact that many children and parents received psychological support. Even though the outcomes were not deeply negative and pervasive at the areas we looked into, we want to stress the importance of diagnostic assessment and the timely start of an evidence-based treatment when it appears necessary. We suggest starting with thoroughly assessing what the child and/or parent really needs, instead of only be focusing on trauma treatment.

An additional important implication is the awareness that clinical PTSD symptoms in parents will not remit naturally and therefore diagnostic assessment and evidence-based treatment are of high importance. The manifestation of PTSD in parents seems to be more persistent than the negative emotional reactions following the disclosure of the CSA. Thus, mental health professionals ought to keep in mind that a client might have underlying PTSD symptoms even though the negative emotional reactions reduce.

For longitudinal studies, attrition rate remains a problem. This was also a considerable threat for our study, as the group is small and further recruitment is not possible as this study is part of a specific sexual abuse case. It was briefly mentioned that the attrition rate in our study is low; parents of five children dropped out after the first time point and parents of two children dropped out after the fourth time point. There are several points that could be related to the low attrition rate; firstly, we have tried to keep the experienced researchers involved in this study as consistent as possible and the number of researchers as limited as possible. Moreover, the researchers involved in this study are all educated as child psychologists or child experts and experienced as researchers or clinical workers in the trauma field. Secondly, we have chosen not to let interns, research assistants or other external parties to be involved in contact with the parents. Thirdly, in contact with parents, we have tried to be as cautious and considerate of privacy matters as possible. Fourthly, the annual appointments with parents not did not only serve scientific purposes, but we also used it as part of the aftercare for parents and their children. Lastly, we believe that maintaining close ties with parents, being flexible, and treating them as individuals instead of subject-numbers, might have helped in the continuous participation.

To conclude, the current study shows the importance of long-term monitoring for children and their parents, as this study shows that despite psychological support, children and their parents still show psychological problems until 8 years after the abuse. This urges professionals to be aware for not only trauma symptoms, but atypical symptoms as well. Overall, considerable insight has been gained with regard to the long-term course of negative psychological sequelae of children who are sexually abused at a very young age and their parents. We believe it is clear that every single victim is one too much and we want to stress that CSA leaves scars, even on the youngest victims.

This research has raised many questions in need of further study. Our data collection is ongoing to examine whether the observed negative psychological sequelae remain stable, keep decreasing or fluctuating, and determine the interaction between outcomes for children who were sexually abused at a very young age and the outcomes for their parents. The following topics are deferred to our future work and will be discussed in separate papers; sexual behavior, attachment, and the influence of disclosure.

As a final point, we have been talking in numbers and percentages, but let us keep in mind that we are talking about individuals. We do realize the tremendous impact of CSA on each and every family. The authors sincerely acknowledge the valuable contributions of the parents and children who participated or are still participating in this study. The authors also wish to thank the four mental health professionals: NS, IH, KZ, and EB for categorizing the psychological support data.

This study was made possible from the financial support of the Public Health Service of Amsterdam, the University Medical Centers of Amsterdam, the Ministry of Security & Justice, and the Ministry of Health, Welfare and Sport of the Netherlands.

## Summary

Although the literature on Child Sexual Abuse (CSA) has increased over the past years, still little is known about CSA in very young children. This study examined the long-term course of psychological outcomes of CSA (PTSD, dissociation and behavioral problems) in children who have been sexually abused in infancy or early childhood. Additionally, we have looked into the outcomes for parents by assessing PTSD symptoms and negative emotional reactions towards the sexual abuse of their child. We examined the outcomes for five consecutive years in a sample of children (n = 45) who were sexually abused at a very young age (0–3) and their parents (n = 42), included in the Amsterdam Sexual Abuse Case-study (ASAC). Questionnaires were administered to the parents. We observed low percentages of PTSD and dissociation in children at each time point. Dissociation symptoms seemed to decrease over time. Contrastingly, we found that PTSD symptoms remained stable over time. However, the number of children exhibiting clinical problem behavior kept fluctuating over the years. For 27% of the parents, the sexual abuse in their child led to clinical PTSD symptoms. PTSD in parents fluctuated gradually over the years, whereas negative emotional reactions decreased. Negative sequelae in the aftermath of CSA appear to apply to children who were sexually abused during infancy or early childhood as well, even if, at the time of the abuse their cognitive capacity was limited. Secondly, it is becoming clear that symptoms within children vary in atypical problems as behavioral problems, rather than only in trauma symptoms. Lastly, results indicate the importance of long-term monitoring for children as well as their parents. Further longitudinal and multi-informant studies are needed to elucidate these results.

## Electronic supplementary material

Below is the link to the electronic supplementary material.

Supplementary file1 (DOCX 38 kb)

## Abbreviations

ASAC: Amsterdam sexual abuse case
CSA: Child sexual abuse
