# Supplementary material for: The Amsterdam Sexual Abuse Case: What Scars did it Leave? Long-Term Course of Psychological Problems for Children Who have been Sexually Abused at a Very Young Age, and their Parents
Source: Child Psychiatry Hum Dev. 2020 Oct 3;52(5):891–902. doi: 10.1007/s10578-020-01067-5 (PMC8405499; doi:10.1007/s10578-020-01067-5)
Supplement: Supplementary file 1 — Supplementary file1 (DOCX 38 kb) [file 10578_2020_1067_MOESM1_ESM.docx]

*Measures*

To assess psychopathology in children, the following instruments were used:

- The Dutch version [1] of the Children’s Revised Impact of Event Scale, Parent Version [CRIES-13; 2] was used to measure Posttraumatic Stress Disorder (PTSD) symptoms in children. The 13 items asses symptoms of intrusion, avoidance, and hyperarousal. The total score indicates the level of severity of the posttraumatic stress response, where a total score of 30 or higher was used as clinical cutoff [3]. Parents reported on the frequency with which their child has experienced these symptoms on 4-point Likert scale (0 = not at all, 5 = often). Based on national and international research, the validity and internal consistency of this questionnaire were found to be from good to excellent [3]. The internal consistency in the current study, using Cronbach’s alpha ranged from α = .89 to .91 over the five time points, for the total score.
- The Child Dissociative Checklist [CDC; 4], Dutch version [5] was used to measure dissociation symptoms in children. This questionnaire consists of 20 items. Parents reported about their children by indicating dissociation symptoms on 3-point Likert scale from 0 (not true) to 2 (very true). The total score could range from 0 to 40. Scores of 12 and higher were considered clinical, indicating pathological dissociation. The questionnaire has a good construct validity and excellent internal consistency [4]. The internal consistency in the current study using Cronbach’s alpha was α = .64 to .73 over five time points.
- The Child Behavior Checklist 1½-18 [CBCL; 6], Dutch version [7] was used to determine the level of internalizing and externalizing behavioral problems in children. Parents reported on the psychological function and behavioral problems of the child. The questionnaire consisted of 100 (for children from 1½-5 years old) to 113 items (for children from 6-18 years old) on 3-point Likert scale from 0 (not true) to 2 (very true or often true). T-scores were calculated for each subscale. A T-score of 65 or higher indicates problem behavior at clinical level. In our study, we have focused on the subscales Total problem behavior, Internalizing- and Externalizing problem behavior. Total problem behavior indicates the sum of all items excluding the items about asthma and allergy. The subscale Internalizing problem behavior refers to the sum of scores of the Anxious/Depressed, Withdrawn and Somatic problems syndrome profiles. The subscale Externalizing problem behavior refers to the sum of scores on Aggressive and Delinquent syndrome profiles [8]. The reliability and validity of this questionnaire are found to be adequate to good [9]. The internal consistency of the current study using Cronbach’s alpha ranged from α = .95 to .98 during the first two time points (as it is not applicable from time point 3 due to the age of the children) for CBCL 1½ - 5 years old, and ranged between α = .95 and .96 over the five time points.

To assess psychopathology in parents, the following self-report instruments were used:

- The Impact of Event Scale-Revised [IES-R; 10], Dutch version [11] was used to determine PTSD symptoms in parents. This questionnaire consists of 22 items. Parents reported about themselves on 5-point Likert scale from 0 (not at all) to 4 (extremely). The total score could range from 0 to 88, where a total score of 23 or higher was used as clinical cutoff. The reliability and validity of this questionnaire range from adequate to good [12]. The internal consistency in the current study using Cronbach’s alpha was between α = 89 and α = .97 over the five time points.
- The Parent Emotional Reaction Questionnaire [PERQ; 13], Dutch version [14]
  was used to measure the negative emotional reactions of parents towards the sexual abuse of their child. This questionnaire consists of 15 items examining the reactions fear, guilt, anger, embarrassment, and feeling upset towards the abuse of their child, reported on 5-point Likert scale from 1 (never) to 5 (always). The total score ranges from 15-75 and results in three subscales: distress, shame, and guilt. This questionnaire is found to have a good reliability and validity [13]. For the analyses, item 15 was left out in order to be able to compare the outcomes, as suggested by Holt, Cohen [15]. The internal consistency in our current study using Cronbach’s alpha ranged from α = .74 to .84 over the five time points.

**References**

1. Verlinden, E., M. Ollf, and R.J.L. Lindauer, *Dutch version of the Children’s Revised Impact of Event Scale for very young children (CRIES-13, parent version)*. 2005.

2. Foundation, C.a.W. *The Children's Revised Impact of Event Scale (13): CRIES-13*. 1998 January 23, 2017].

3. Verlinden, E., et al., *Characteristics of the Children's Revised Impact of Event Scale in a clinically referred Dutch sample.* Journal of Traumatic Stress, 2014. **27**(3): p. 338-344.

4. Putnam, F.W., K. Helmers, and P.K. Trickett, *Development, reliability, and validity of a child dissociation scale.* Child Abuse Negl, 1993. **17**(6): p. 731-41.

5. Hartveld, G. and M. Janssen, *Nederlandse vertaling van de Child Dissociative Checklist (CDC).* Amsterdam: VU University Amsterdam, 1992.

6. Achenbach, T.M., L. Dumenci, and L.A. Rescorla, *DSM-oriented and empirically based approaches to constructing scales from the same item pools.* J Clin Child Adolesc Psychol, 2003. **32**(3): p. 328-340.

7. Verhulst, F.C., J. Ende, and H.M. Van der Koot, *Handleiding voor de CBCL/4-18*, ed. J. Ende. 1996, Rotterdam: Sophia Kinderziekenhuis.

8. Achenbach, T.M., *Manual for the Child Behavior Checklist 4-18 and 1991 Profiles*. 1991, Burlington: VT: University of Vermont, Department of Psychiatry.

9. Koot, H.M. and F.C. Verhulst, *Prevalence of problem behavior in Dutch children aged 2‐3.* Acta Psychiatrica Scandinavica, 1991. **83**: p. 1-37.

10. Weiss, D. and C. Marmar, *The Impact of Event Scale - Revised*, in *Assessing Psychological Trauma and PTSD*, J. Wilson and T. Keane, Editors. 1997, Guilford Press: New York, NY. p. 399–411.

11. Mouthaan, J., et al., *Comparing screening instruments to predict posttraumatic stress disorder.* PLoS One, 2014. **9**(5).

12. Mouthaan, J., et al., *Comparing screening instruments to predict posttraumatic stress disorder.* PLoS One, 2014. **9**(5): p. e97183.

13. Mannarino, A.P. and J.A. Cohen, *Family-related variables and psychological symptom formation in sexually abused girls.* Journal of Child Sexual Abuse, 1996. **5**(1): p. 105-120.

14. Diehle, J., M. Abrahamse, and R.J.L. Lindauer, *Dutch translation of the Parent Emotional Reaction Questionnaire (PERQ)*. 2013, Amsterdam, The Netherlands: De Bascule. Available upon request.

15. Holt, T., J.A. Cohen, and A. Mannarino, *Factor structure of the parent emotional reaction questionnaire: analysis and validation.* European journal of psychotraumatology, 2015. **6**(1): p. 28733.
